# Supplementary material for: Are carbon emissions trading and green financial instruments synergistic? -Comprehensive quantitative research based on content analysis
Source: PLoS One. 2024 Mar 7;19(3):e0298601. doi: 10.1371/journal.pone.0298601 (PMC10919601; doi:10.1371/journal.pone.0298601)
Supplement: S1 Data — (ZIP) [file pone.0298601.s001.zip › Data/地级市Z_F工作报告.zip!/╜¡╬≈/╛┼╜¡╩╨/└┤╫╘╣1⁄2╓┌║┼í╛┬φ┐╦╩2╛▌═°í┐_2014.docx]

各位代表：

　　现在，我代表市人民政府，向大会报告工作，请予审议，并请各位市政协委员和列席会议的同志提出意见。

　　一、2013年工作回顾

　　过去的一年，在省委、省政府和市委的正确领导下，全市上下深入贯彻党的十八大和十八届三中全会精神，全面落实省委十三届七次全会的战略部署，始终围绕“九江争得应有地位、人民过上小康生活”的目标，牢牢把握“两区互动、强工兴城”的工作重点，深入推进沿江开放开发和昌九一体，有效克服了经济下行的压力，经济社会保持了良好的发展态势，完成了年初确定的各项目标任务。

　　（一）主要经济指标跃上新台阶。全市完成生产总值1601.73亿元，增长10.4%；财政总收入280.2亿元，增长26.9%，总量全省第二，增速全省第一；公共财政预算收入176.15亿元，增长24.2%，县县财政收入过10亿元，新增4个过20亿元。500万元以上项目固定资产投资1507.78亿元，增长25%。社会消费品零售总额435.63亿元，增长13.8%。居民消费价格总水平上涨2.6%。城镇居民人均可支配收入22504元，增长10.7%；农民人均纯收入8805元，增长13.1%。实际引进市外亿元以上项目资金299亿元；实际利用外资123107万美元，增长24.6%；完成外贸出口403370万美元，增长14.4%。

　　（二）沿江开放开发取得新成效。全省聚焦沿江开放，全市聚力沿江开发，沿江板块的承载力更强，产业集聚度更高，引领全市、服务全省的作用更大。沿江区域实施亿元以上重大产业项目128个，累计完成投资558.5亿元；实施5000万元以上重大基础设施项目63个，累计完成投资170.3亿元。长江二桥、赤码大道、彭湖大道和城西、城东港区疏港通道建成通车。城东港区公用码头和九鼎、理文等业主码头建成运营。500千伏马回岭、220千伏都昌蔡岭扩建、110千伏姑塘变电站和共青智能电网等重点输变电工程建成投运。九江电子口岸建设、关港联网与运用系统建设加快推进。沿江港口货物吞吐量超过6000万吨，增长30%；集装箱18万标箱，增长12.5%。全面贯彻省委省政府“做大九江、昌九一体”的战略部署，制定出台了“一个决定、两个方案”。启动了昌九一体化和共青先导区发展总规及专项规划编制，制定出台了共青先导区总体方案和实施意见。昌九高速“四改八”试验段等重大基础设施快速推进，昌九城际车次加密，南昌至永修开通公交，昌九一体化步伐明显加快。

　　（三）工业经济总量实现新扩张。完成规模以上工业主营业务收入3859.74亿元，增长22.94%；工业增加值784.83亿元，增长13.3%；利税总额411.52亿元，增长43.34%，工业对经济增长的主导作用更加凸显。十大产业实现增加值653.2亿元，占全市比重83.2%；实现利润155亿元，增长35%；五大新兴产业主营业务收入突破1000亿元，增长35%。净增规模以上工业企业163户，达1009户；新增利税过亿元企业21户，达58户；新增主营业务收入过亿元企业58户、过10亿元企业14户，分别达646户、45户；13家企业获“江西名牌”称号，6家企业获首届“市长质量奖”。完成园区基础设施建设投入169.91亿元，增长6.69%；全市园区主营业务收入2858.21亿元，增长20.94%，新增百亿园区3个，实现园园过百亿，成功创建四个省级产业基地。民营经济快速发展，完成销售收入2800亿元，增长24.8%，中小企业达3万余户。一批重大产业项目快速推进，理文造纸、理文化工、龙达化纤、亚东水泥五六期、青岛啤酒、华祥线路板、杭氧制氧机、富美家装饰、九江金鹭硬质合金、喜得龙体育用品、中浩纺织等一批项目建成投产；九江石化油品质量升级、恒生大化纤、蓝星星火有机硅二期、德鑫纺织二期等一批项目快速推进；神华煤电、镍基复合合金、九宏新材料、攀森新材料三期、中船装备产业园、中粮百亿产业园等一批项目开工建设。

　　（四）城市面貌发生新变化。八里湖新区基本成型，功能不断完善。全年完成政府性投资50亿元，带动社会投资150亿元。市民服务中心、文化艺术中心、市委党校新校区、东组团安置小区、前进西路改造等15个项目建成；通岭大道、九派诗廊、房产交易中心、移动通信中心等23个项目快速推进；引进落户开发性项目20个，中航城、绿地ICC大都会、水岸莲华、宇龙湖畔花园等项目开工建设。老城改造步伐加快，浔阳区“2111”工程全面启动，封缸酒厂、孔家洼、战备路、莲花池等4个片区拆旧动迁基本完成；滨江生态环境改造和琵琶亭公园建设基本完工；长虹东大道建设加快推进，庾亮南路、浔阳路改造全面完工。庐山区浔南城市客厅、五里片区等组团开发加速推进，九莲北路建成通车，濂溪大道延伸线、学院路二期等进展顺利。九江经济技术开发区八里湖北大道和沙阎北路路桥工程、港城大道、余水路、港兴路建成通车。积极实施城区水系大连通工程，新开河东堤整治加固和十里河调蓄水源工程开工建设，白水湖、浔南片区、濂溪片区的截污和老城区污水管网等项目加快推进。新增公交车辆100辆，新开通公交线路4条。以“城市管理年”活动为抓手，违法建设、建筑工地、垃圾广告、户外广告等专项整治成效明显；省级森林城市创建成功，数字化城市管理系统加快建设。九江绕城高速、都九高速（星子至九江段）等项目快速推进，105国道九江段竣工通车，大九江同城步伐不断加速，县城建设亮点纷呈，城市品位不断提升。

　　（五）“三农”工作迈出新步伐。粮食总产32亿斤，大旱之年实现“十连丰”；新增土地流转14.7万亩，新建千亩以上特色农产品基地60个，新增“三品一标”69个；新上亿元以上农业项目38个，新增年销售收入500万元以上农业龙头企业58家、省级龙头企业15家、国家级龙头企业1家。新增农民专业合作社759家，注册登记家庭农场135家，农机专业合作社80家。修水辽南线、都昌都中线竣工通车，全市改造农村公路1500公里。全面完成2座中型水库、200座一般小（Ⅱ）型水库除险加固和13条中小河流治理防洪主体工程；25万农村人口饮水得到有效改善；新增、恢复灌溉面积9万亩，改善灌溉面积18万亩，建设高标准农田6万亩。40个重点乡镇、87个中心村的镇村联动工作全面铺开，修水县偏远山区“整村移民搬迁进城镇”模式在全国推广。802个新农村建设点村庄整治、新社区建设和清洁工程全面完成，九景、永武、彭湖高速公路沿线村庄整治成效明显。乡镇“两所一队”全面覆盖，“两违”建设得到有效遏制，农民建房管理不断加强。鄱阳湖采砂管理更加规范。完成造林绿化30万亩，新建绿道274公里，新增森林乡镇42个、森林村庄119个、森林公园24个，新增国家级生态乡镇8个、省级生态乡镇7个、省级生态村10个，乡村生态环境明显改善。

　　（六）第三产业实现新发展。庐山品牌国际化迈出新步伐，联合国教科文组织《世界文化景观—庐山宣言》正式发布，庐山西海获评国家水利风景区，星子县被评为“美丽中国十佳旅游县”，武宁县被列为全省首批旅游综合改革试点县和旅游产业园区县。庐山大林路、牯岭街、山南公路和庐山西海司马码头、武宁西海燕码头等景区基础设施完工，东林大佛、中华贤母园等景点建成开放；庐山天然气上山、庐山新城、中信庐山西海度假区、山水菁华养生养老中心等项目顺利推进。全市新增4A级景区2个、4A级乡村旅游点1个，“跟着课本游九江”、“万名台胞游九江”成为旅游新模式，大型音舞诗画剧“春江花月夜”、武宁西海湾景区夜游等成为旅游新亮点。全年接待旅游总人数5613万人次，增长40%；旅游总收入402亿元，增长32.6%。商贸物流加快发展，现代综合大市场、大润发商业广场、21世纪家居中心、华联生活广场、欧洲风情一条街等项目建成，宏大五金、联盛十里老街、京九国际商贸城等项目快速推进，新增商业面积250多万平方米；南方粮食交易市场和鄱湖珠贝网等第三方电商交易平台发展迅速，共青城市被确定为省级电子商务示范基地。金融保险、家政服务、中介服务、货运代理、广告设计等新兴服务业加快发展，全市金融机构本外币存贷款余额1759.04亿元和1056.05亿元，分别增长17.7%、14.1%；中信银行九江分行挂牌营业，全市金融机构达17家；通过“助保贷”、“财企惠贷通”等方式，为小微企业融资238.7亿元，有效缓解了企业融资压力。

　　（七）社会建设取得新进步。全市用于民生方面的财政支出达到210.6亿元。新开工建设保障性住房93122套，新竣工33892套，其中中心城区新开工49608套、新竣工10884套，为1.79万户发放租赁补贴4514万元。新增城镇就业6.33万人，新增转移农村劳动力6.01万人，发放小额担保贷款7.5亿元，购岗安置4000人。新农合参合率98.4%，职工基本医疗保险参保率稳定在95%以上。城市低保保障标准提高到每人每月400元，农村低保保障标准提高到每人每月200元，累计发放低保金5.3亿元，改造提升农村敬老院52所。“重生行动”、“明天计划”等助医助学项目成效明显，“光明·微笑”工程、“儿童白血病和先天性心脏病”、贫困患者尿毒症、重性精神病免费救治有效实施。投入资金5.54亿元，在395个贫困村实施扶贫开发，搬迁深山区、库区、农村污染区移民1.4万人。新建改造农贸市场4个，创建“放心粮油”示范点65个，新建社区便民蔬菜直销店20家。13个县（市、区）全部通过全国科技进步县考核，新增高新技术企业13家，专利授权量突破1000件。全市财政教育支出63.2亿元，市本级支出11.9亿元，新、改、扩建校舍面积30万平方米，双峰小学庐山区校区、九江学院八里湖校区、出口加工区学校等项目竣工，九江职业大学、九江职业技术学院和江西财经职业学院等新校区建设快速推进，学前教育三年行动计划全面启动，补充教师1315名。在全国率先开展“区域医疗联合体”改革试点，“先看病后付费”诊疗模式全面铺开，新扩建医疗卫生机构161个、面积12万平方米，招聘卫生技术人员654名，市一医院康复保健楼、全科医生培训基地投入使用，三级卫生服务网络进一步健全。县级“三馆”、乡镇综合文化站、农家书屋三级公共文化设施基本完善。全民健身运动蓬勃开展，九江籍选手在第十二届全运会上摘得两块金牌。人口计生信息化平台建设全面启动，流动人口服务管理体系基本建成，人口自然增长率7.22‰。全力创建全国食品药品安全示范区，食品药品安全形势进一步好转。市政府民声直通车全年受理和办理群众意见、建议、诉求7781件。信访和安全生产形势总体平稳，应急处置能力进一步提升。平安九江、法治九江建设加快推进，公众安全感继续位居全省前列，浔阳区荣膺“全国社区戒毒社区康复工作示范单位”称号。积极开展环保专项行动，建成新的城区大气监测体系，完成省政府下达的节能减排任务。财政、户籍、国资监管、投融资体制、事业单位分类和行政审批制度等改革全面推进，省属改制企业社区移交工作全面完成。自觉接受人大及其常委会依法监督和政协民主监督，办理市人大代表建议211件、市政协提案445件。全面落实中央“八项规定”，坚决反对“四风”，廉政建设进一步加强。统计、审计、海关、海事、检验检疫、气象、水文、盐业、通讯、邮政、无线电管理、档案、国安、保密、编制、地方志、对台、外事侨务、民族宗教等工作得到新加强；国防动员、民兵预备役、双拥创建、人民防空、防震减灾、消防等工作取得新成绩；妇女儿童、老龄、残疾人、红十字和慈善等事业实现新发展。

　　各位代表，回顾一年来的政府工作，成绩来之不易！我们深切地感受到：所有成绩的取得，得益于省委、省政府和市委的坚强领导，得益于“两区互动、强工兴城”的正确思路，得益于市几套班子和全市人民的共识共为。在此，我代表市人民政府，向各位人大代表、政协委员，向全市人民，向离退休老同志，向各民主党派、工商联、无党派人士、各人民团体，向驻浔人民解放军、武警官兵和公安民警，向中央和省驻浔单位，向所有关心、支持和参与九江建设的朋友们，表示衷心的感谢并致以崇高的敬意！

　　各位代表，在看到成绩的同时，我们也清醒地认识到：经济总量不大、发展不足仍是我市的主要矛盾，加快发展、做大九江的任务还十分艰巨；产业水平不高，企业创新能力不强，产业升级和节能减排的任务还十分繁重；城乡居民收入偏低，部分群众生活比较困难，保障和改善民生、加快小康步伐的任务还十分紧迫；行政效能有待进一步提高，发展环境还需进一步优化。对此，我们将高度重视，采取有效措施，认真加以解决。

　　二、2014年工作安排

　　2014年是全面贯彻落实党的十八届三中全会精神、全面深化改革的开局之年，是实施“十二五”规划的攻坚之年，是决战大工业、实现大跨越的首战之年。做好今年的政府工作，意义重大，任务艰巨。

　　今年政府工作的总体要求是：深入贯彻党的十八大和十八届三中全会精神，全面落实省委十三届七次、八次全会的战略部署，以做大九江为目标，大力推进沿江开放开发和昌九一体化，决战大工业、打造大门户、推进大统筹、改善大民生、实现大跨越，为九江争得应有地位、人民过上小康生活而努力奋斗。

　　全市经济社会发展主要预期目标是：生产总值增长11%；财政总收入增长15%；固定资产投资增长20%；社会消费品零售总额增长15%；居民消费价格涨幅控制在3.5%左右；城镇居民人均可支配收入和农民人均纯收入均增长12%；人口自然增长率控制在7.45‰以内；完成节能减排任务。

　　为实现上述目标，重点抓好七个方面的工作：

　　（一）着力推进昌九联动，优化区域布局做大九江

　　全面落实省委、省政府“做大九江”的重大战略部署，积极策应长江中游城市群和鄱阳湖城市群建设，充分放大九江的独特优势，加快构建“沿江挑大梁、沿路做支撑、县区为依托”的T型发展格局，努力肩负起“昌九一体、龙头昂起”的光荣使命。

　　有序推进昌九一体化。按照“规划一体化、基础设施一体化、公共服务一体化和产业互补对接”的总方向，争取上级支持，主动对接南昌，全力把九江打造成支撑江西经济崛起的“双核”之一。加快重大基础设施建设，开工建设昌九高速全线“四改八”、修平高速、昌九大道、长江大桥改造等项目，继续推进九江绕城高速、都九高速等项目；协调推进彭湖高速对接安徽高速路网建设，开工建设九景衢铁路、武九客专，做好合九客专前期工作，逐步形成九江到南昌、杭州、武汉、长沙、合肥五个省会城市直达快捷交通网络；积极对接昌北机场，完善配套设施，逐步把九江机场建成国内重要的支线旅游机场。加快放大公共服务“同城效应”，取消昌九之间移动通讯漫游费和固定电话长途费，逐步实现昌九两地户籍、金融、公积金、社会保障等公共服务一体化。

　　有力推进沿江开放开发。充分发挥黄金水道、深水岸线和港口码头的独特优势，继续把沿江板块作为做大九江的主战场。进一步完善基础设施，按照《九江沿江港口码头规划》，合理确定港口功能定位和发展方向，规划推进长江岸线综合工程，建好公用码头，用足业主码头，优化港口资源配置，提高岸线利用效率，支持上港集团在九江加快发展，启动彭湖湾港区建设，增强九江港综合服务能力；启动城东、城西、彭湖等板块的铁路专用线建设，改造升级湖口、彭泽长江大堤，加快城西港区至赤湖、湖牛线台山至定山段改造等一批疏港通道建设，规划启动九江至湖口湖底隧道前期工作，加快形成水、铁、公、机为一体的集疏运体系；开工建设500千伏石钟山至洪源线路，220千伏码头、沙城扩建和110千伏德化、沿浔、长坪、太平等输变电设施，实施中西部电网优化工程。进一步优化空间布局，继续推进城西港区二期开发，加快建设一批临港新城，积极申报九江综合保税区，打造沿江港区、物流园区、工业园区和临港城区“四区一体”的发展布局。进一步壮大临港产业，着力推进九江石化油品质量升级、高钴氢氧化镍、西矿铜业、瑞智机电产业园、200万吨焦化、中部红木产业园、海底电缆、赤湖皮革工业城等项目建设，带动配套产业集聚发展，打造主业突出、特色鲜明、链条完整、支撑有力的临港产业集群。进一步加快对接互动，促进板块、县区、江路间的联动、错位、互补发展。力争沿江区域完成固定资产投资1200亿元、规模以上工业主营业务收入突破3000亿元。

　　有效推进共青先导区建设。全面实施《关于建设鄱阳湖生态经济区共青先导区的意见》，用活用好共青城市设区市的经济管理权限，兴起新一轮建设发展高潮。加快推进南湖新区“四横四纵”路网建设，开工建设城南高速互通及连接线、共青城至星子温泉快速通道等基础设施项目。加快推进中通金域云计算中心、共青电子商务学院、手机产业园、国际服装城、鸭鸭创新基地、农业示范区等项目，逐步构建电子商务、终端智能、服装创意、动漫软件、观光农业等五大特色产业基地。加快共德永融合发展步伐，推进园区调区扩区，着力培育电子信息、现代轻纺和新能源新材料三个百亿级绿色产业，逐步把先导区打造成为绿色发展的示范区、新型城镇化的先行区、体制机制创新的试验区、昌九一体化的重要支点。同时，按照“权力要下放、政策要松绑、服务要到位”的要求，加大分类指导力度，进一步完善激励机制，健全考核体系，促进县域经济竞相发展、错位发展、特色发展。

　　（二）全力决战大工业，扩张经济总量做大九江

　　紧紧围绕“三年翻一番、五年过万亿”的目标，以最大的决心和行动强攻工业，既要做大总量，又要提升质量，规模以上工业主营业务收入确保4800亿元、力争5000亿元，增加值900亿元、利税总额400亿元。

　　培育产业龙头。通过技改扩能、优化升级，千方百计扩大企业规模，壮大产业群体。支持九江石化、九江钢厂、九江电厂、蓝星星火有机硅、昌铃汽车、巨石玻纤等骨干企业技改扩能，推动理文化工、理文造纸、龙达化纤、大唐化学、九宏新材料、攀森新材料、青岛啤酒等企业尽快建成投产、达产达标。以大项目为依托，加快配套一批中小企业，推进产业延链互补，促进大、中、小企业协同发展，形成龙头企业顶天立地、配套企业铺天盖地的产业发展格局。力争新增规模以上企业100户，新增主营业务收入过亿元企业100户以上、过10亿元的10户以上、过20亿元的6户以上。

　　促进产业集聚。围绕打造石油化工、钢铁有色冶金、现代轻纺、电力新能源四大千亿产业集群，抓好九江石化800万吨油品质量升级和石化产业园建设，推进40万吨有机硅单体及24万吨下游系列产品等项目建成投产，力争石油化工产业主营业务收入突破700亿元；推进九钢与方大特钢的“城市钢厂搬迁工程”，加快镍基复合合金、城门山铜矿三期等项目建设，推进九江金鹭硬质合金等企业达产达标，大力发展以钨、铅、锌、铜等有色金属为主的精深加工，力争钢铁有色冶金产业主营业务收入突破500亿元；推进恒生大化纤、德鑫纺织等项目建设，促进棉纺产业产品结构调整，力争现代轻纺产业主营业务收入达750亿元；推进神华煤电一体化、九江电厂四期、光伏及风力发电等项目建设，力争电力新能源产业主营业务收入达200亿元。推进北汽重组昌河铃木、志高空调三期、中科鑫星新材料、中船装备产业园、中粮百亿产业园等项目建设，加快新能源、新材料、电子信息、先进装备制造、绿色食品等新兴产业发展，力争战略性新兴产业实现主营业务收入1500亿元。

　　优化产业平台。进一步优化园区平台，重点抓好园区的水、电、路、污水处理等设施建设，加快融资、用地、用工等服务平台和标准厂房建设，推进园区调区扩区，完善园区生产和生活性配套，不断提升工业园区的承载力，着力把九江经济技术开发区、湖口金砂湾工业园、城东港区、瑞昌工业园、永修云山经济开发区打造成千亿园区。进一步优化物流平台，完善港区功能配置，加快港城、港工、港贸联动，大力发展大宗商品物流和货物仓储业，着力打造“亿吨大港”。进一步优化开放平台，充分发挥海关、口岸、边检、海事、检验检疫等部门的作用，加快完善口岸大通关应用系统，提升通关效率和外贸便捷度，降低企业综合商务成本。

　　做好产业招商。围绕打造产业承接高地，优化发展环境，强化以情招商，抢抓“央企入赣”的机遇，着力引进一批大央企、大省企；顺应产业加快转移的大势，着力引进一批大外企、大民企。围绕聚集上下游产品、延伸产业链条、降低综合配套成本、培育优势支柱产业，强化龙头延链、配套补链，着力引进一批配套型、服务型、延链型项目，加快形成产业集聚、企业集群发展的格局。围绕十大主导产业，强化以商招商，充分发挥产业协会、企业商会作用，着力引进一批龙头型、基地型产业项目。

　　（三）加快推进新型城镇化，打造门户城市做大九江

　　积极顺应国家构筑长江中游城市群的大势，主动融入“中四角”的城市发展格局，发挥依山傍水、滨湖临路的独特优势，加快建设大九江都市区，努力把九江打造成为宜居宜业宜游的省域门户城市、长江经济带重要节点城市。

　　健全大九江都市区规划体系。按照“生产空间集约高效、生活空间宜居适度、生态空间山清水秀”的要求，围绕构建以中心城区为核心、中等城市为支撑、重点集镇为节点的大九江都市区，启动编制大九江都市区总体规划和专项规划，形成覆盖城乡、相互衔接、科学合理的城乡规划体系。以规划为龙头，联动“四县一山”，加快完善新城区、改造老城区、建设拓展区、构建都市区，鼓励支持共青城、修水、瑞昌、都昌向中等城市发展，打造一批沿江特色产业集镇、沿路物流节点集镇、滨湖生态旅游集镇、山区边贸中心集镇。

　　完善提升八里湖新区。按照“行政文化中心、商业居住胜地、休闲观光乐园”的功能定位，加快实现由政府投资为主向社会投资为主转变、基础设施为主向公共服务为主转变、环境建设为主向聚集人气为主转变。开工建设兴城南大道、八里湖中央商务区、九江国际金融广场、中奥广场、八里湖宾馆、蛟滩污水处理厂等一批项目；续建推进通岭大道、绿地ICC大都会、中航城、移动通信中心、九江国际湾、八里湖公园等一批项目；建设完成市儿童医院、市民中心公园等一批项目，进一步完善新区公共服务功能，提升新区人气、财气和商气。

　　改造提升老城区。加大棚户区改造力度，用两年时间在中心城区改造棚户区500万平方米，其中庐山区300万平方米，浔阳区、九江经济技术开发区各100万平方米。浔阳区按照“分片改造、整体提升”的思路，推进京九市场搬迁、战备路安置区建设等项目，启动考棚、黄土坡、面粉厂、啤酒厂等片区改造动迁；庐山区按照“整区推进、整体提升”，加快实施五里片区、十里片区改造；开发区重点实施三马路片区改造。加大边街小巷改造力度，按照“市区联动、属地负责”的原则和“水通、路平、灯亮、线齐、墙美”的标准，用两年时间基本完成老城区边街小巷改造。加大城区干道建设改造力度，推进长虹东大道建设和浔阳西路沿线、长虹立交桥改造提升，启动琴湖大道（南段）、芳兰大道、滨江西大道（西段）建设；推进高速公路九江南、荷花垄、白水湖互通口改造提升，规划启动九江火车站、庐山站综合改造，提升九江“城市之门”的窗口形象；加强市政设施管护，完善城区水气电讯等地下管网配套设施，启动第四自来水厂建设；开工建设白水湖环湖公园，启动市动物园搬迁；加大便民设施建设改造力度，新建、改造提升一批农贸市场、社区用房、公共厕所等服务设施；加快公共停车场建设，优化交通组织，推进城区公交化和城郊客运一体化进程；加大历史文化街区、文物保护单位、非物质文化遗产保护力度，推进九江茶市、庾亮南路历史文化街区等项目建设，挖掘文化内涵，留住城市记忆，彰显城市个性。

　　加强城市管理。坚持以人为本的和谐城管理念，巩固和扩大“两违”建筑、“两声三乱”等整治成果，进一步加强交通秩序、小区物业、广告店招、门前“三乱”、建筑工地等整治管理；坚持“花钱买管理，不花钱养人”，建立健全城市管理长效机制，做好新建公共服务场所、设施管理跟进工作；加强城区环境保护和生态建养，抓好机动车污染防治，减少道路及施工扬尘，严控二氧化硫、氮氧化物等工业废气排放，完善污水处理和大水系联通设施，加强城镇污水处理厂和脱硫设施运行监管；弘扬生态文明，推进可持续发展实验区建设，积极创建国家森林城市，守护好九江的青山、绿水、蓝天。

　　（四）扎实推进“三农”工作，夯实发展基础做大九江

　　坚持工业反哺农业、城市支持农村和多予少取放活方针，始终把“三农”工作牢牢抓住、紧紧抓好，让农业成为有奔头的产业，让农民成为体面的职业，让农村成为安居乐业的美丽家园。

　　发展现代农业。促进粮棉油等大宗农产品稳产增产，保障粮食安全；培育新型农业经营主体，鼓励和支持土地承包经营权向专业大户、家庭农场、农民合作社有序流转，促进规模化经营；完善农业社会化服务体系，加强基层农技推广、动植物疫病防治网络建设，落实农产品安全属地管理责任；加强农田水利基本建设，全面修复505座（处）损毁水利工程设施，完成144座一般小（Ⅱ）型水库除险加固及渠系配套，恢复和新增灌溉面积9万亩，改善灌溉面积18万亩，建设高标准农田7万亩，推进气象现代化建设，新建6个水文测站，提高农业综合生产能力；加强农产品加工体系建设，大力发展农产品加工园，培育一批农业龙头企业，新增规模以上农业企业20家。

　　促进农民增收。在巩固传统产业的基础上，培育壮大水产、蚕桑、畜禽、油茶、蔬菜等优势特色产业，加快发展苗木花卉、森林旅游等环境友好型农业，不断调优农业产业结构，力争新扩千亩以上示范基地60个，新增省级以上农产品商标名牌和“三品一标”70个，做强“庐山云雾”、“修水宁红”茶叶品牌，努力增加家庭经营性收入；加强农民技能培训，增强农民工就业能力，新增转移农村劳动力5万人，促进农村剩余劳动力向非农产业转移，逐步实现农民就地市民化，努力增加农民工资性收入；深入推进农村综合改革，落实各项强农惠农富农政策，抓好农村集体土地和农民宅基地确权登记试点工作，推进农民住房财产权抵押、担保、转让试点，努力增加农民财产性收入。

　　改善农村环境。新增26个重点镇、52个中心村的镇村联动建设，引导10万农民集中居住。加强“山、田、路、水”的综合治理，加强农村公路建设与管养，启动农村小危桥改造和25户以上自然村“村村通”工程，改造小危桥100座、农村公路1000公里，提高农村综合客运服务能力。加强农村生产、生活污染综合治理，全面完成835个新农村建设点“三清七改五普及”。改造农村危房1万户，改建农村卫生厕所1万座。继续开展“修河环保行”专项活动，集中开展长江河道采砂管理综合整治，加快创建全国生态文明示范市，支持武宁创建全国生态文明建设示范试点县。大力开展森林村庄建设，继续实施“森林城乡、绿色通道”工程，完成人工造林22万亩，综合治理水土流失面积30万亩。

　　（五）大力发展文化旅游、商贸物流，繁荣现代服务业做大九江

　　文化旅游是富民兴市的绿色产业，商贸物流是潜力巨大的新兴产业。大力发展文化旅游、商贸物流产业，繁荣现代服务业，既是经济发展的重要成果，更是产业发展的良好环境。

　　放大庐山品牌，发展文化旅游业。庐山是九江的，更是世界的；庐山是九江文化旅游的金字招牌，更是九江走向世界的国际名片。围绕打造“江西旅游发展的重要增长极和全国旅游强市”目标，大力推进旅游资源庐山化、庐山品牌国际化，编制完善以庐山为龙头的《九江市旅游发展总体规划》，构筑以庐山为核心，以长江、鄱阳湖、寻阳古城、庐山西海及周边县（市、区）景区为基点，联动“山、江、湖、城”的大旅游经济圈。创新旅游管理体制机制，组建旅游委员会，加大对文化旅游产业支持力度，促进旅游资源由分散开发向整体开放开发转型、旅游消费由观光旅游为主向休闲度假观光并重、旅游收入由门票经济向综合收入升级。加大旅游招商引资力度，支持区域性旅游整体开放开发，加快推进庐山索道、东林大佛文化景区、中信庐山西海度假区、大千世界·青少年科普教育基地、云居山国际禅修中心、庐山西海国际养生度假区等项目；启动建设庐山综合博物馆、国际庐山·鄱阳湖欢乐园、庐山归宗景区、泰国风情度假村、吴城“赣天下”鄱阳湖国际旅游岛等项目。大力促进文化与旅游融合发展，充分挖掘九江丰富的历史文化，支持发展文化旅游创意产业。强化旅游形象推广，创新旅游营销模式，着力打造智慧旅游平台。加强旅游区域合作，鼓励市内旅游企业积极开拓境内外客源市场。培育发展壮大旅行社，规范旅游市场秩序，提高旅游标准化建设水平。

　　依托区位优势，发展商贸物流业。充分发挥长江水道和国家一类口岸优势，以国际水运、全国铁路和区域公路运输三大通道为依托，加快构建立体化的现代物流体系，把区位交通优势变为商贸物流优势。以“城内大卖场、城郊大市场、城乡大流通”为目标，加快推进禧徕乐国际商贸中心、喜盈门国际商业广场、九江国际皮草商贸城、联盛九龙广场、联盛奥特莱斯商业中心等项目建设，打造一批商业综合体和商品大市场；加快推进十里老街、南海路美食夜市、九莲南路电子商务展销街等规划建设，打造一批特色商业街。继续实施“万村千乡”市场工程，改善农村居民消费环境，不断完善商贸流通体系。

　　繁荣城市经济，发展现代服务业。积极引进银行类、保险类、资本类企业入驻九江，鼓励金融机构推进产品和服务方式创新，优化融资结构和信贷结构，提高直接融资比重，探索中小企业互助银行等新型融资模式和政银企合作新途径，大力推进企业上市，扩大“财企惠贷通”融资试点，促进金融业与产业经济的良性互动，把更多的货币资源优化配置到实体经济。稳步发展餐饮住宿、交通运输、邮电通讯等传统服务业，积极发展科技咨询、广告会展、新闻出版、文化创意、研发设计、服务外包等新兴服务业，规范发展电子商务和社区服务、养老服务、中介服务业，有序发展总部经济、楼宇经济和房地产业，促进城市生产和生活服务业实现新发展。

　　（六）加大改革开放力度，激发动力活力做大九江

　　进一步解放思想，解放和发展社会生产力，解放和增强社会活力，放手让一切劳动、知识、技术、管理、资本的活力竞相迸发，让一切创造社会财富的源泉充分涌流。

　　深化重点领域改革。按照中央和省委、省政府部署，推进行政审批制度改革，全面清理、精简审批事项，拓宽项目审批“绿色通道”，对城建项目、工业项目、民生项目进行流程再造、分类审批，继续实行项目代办制、并联审批制，最大限度地减少审批环节，提高办事效率；深化市场体系改革，加强公共资源交易体系建设，发挥市场在资源配置中的决定性作用，逐步建立城乡统一的建设用地市场，完善土地租赁、转让、抵押二级市场；全面完成市县政府机构改革，推进社会组织直接登记改革，清理、整顿、规范中介组织；做好事业单位分类申报与岗位设置管理工作，推进公务员职务与职级并行改革试点；深化财税制度改革，改进预算管理制度，建立规范统一的部门预算体系和财税信息综合平台，加大部门预算和“三公”经费公开力度；推进国有资产管理改革，稳妥解决国企改革遗留问题，强化政府融资平台监管，加强地方政府性债务管理。

　　扩大对外对内开放。全方位扩大对外开放合作，主动对接上海自贸区，加强与岳阳、咸宁、安庆等地合作，加强与沿江城市跨江联动，拓展区域发展空间。大力发展外向型经济，确保引进省外5000万元以上项目资金580亿元，实际利用外资13.5亿美元，实现外贸出口39亿美元；支持企业对外投资，开展劳务合作，拓展国际发展空间。进一步加大对内开放力度，鼓励支持非公有制经济加快发展，坚持内商外商一视同仁、国企民企平等竞争，放宽市场准入，消除隐性壁垒，引导和鼓励民间资本投入基础设施、公用事业、社会事业等领域，全力支持本土人才大胆创业、在外浔商回归创业、务工人员返乡创业，拓展内生发展空间。

　　加快科技创新步伐。实施创新驱动发展战略，加大财政性科技投入，带动企业科技投入，加快形成多元化的科技投入体系。深化市校合作、校企协作，继续开展“百博挂百企”活动，加强与知名大学、大院、大所的对接合作，推进院士工作站建设，着力突破一批核心关键技术，开发一批拥有自主知识产权和自主品牌的高端特色产品，引进一批重大科技项目，培植一批年销售收入超10亿元的高新技术企业。推动创新资源向企业集聚，支持企业加强研发机构建设，新培育创新型企业30个，实施重大高新技术成果产业化项目20个，建设国家、省和市级协同创新技术研发平台10个，打造国家、省级高新技术产业特色基地和科技园区10个，组建产业技术创新联盟和优势科技创新团队5个。积极对接国家“创新人才推进计划”和“赣鄱英才555工程”，引进一批高层次创新人才。

　　（七）倾力保障和改善民生，加速小康步伐做大九江

　　民生大于天，民心重如地。人民群众对美好生活的向往，就是我们的工作目标。在实际工作中，既考虑政府为群众做什么，又考虑群众需要政府做什么；既关心群众的现实利益，又关注群众的切身感受；既注重群体的普惠民生，又关心个体的特殊困难，不断提高广大人民群众的幸福指数。

　　加大民生保障力度。新开工各类保障性住房7.1万套（户），基本建成8万套；改造城市、工矿、林场、垦区等各类棚户区3.6万户、329万平方米，其中中心城区1.8万户、130万平方米，发放廉租住房租赁补贴1.8万户。新增城镇就业5.42万人，为城乡困难群众购买公益性就业岗位5000个，通过“阳光工程”、“雨露计划”等培训农民工3万人，积极扶持大学生创业，着力破解高校毕业生就业难题。大力改善市民出行条件，中心城区新建公共停车场4个、新增公交车100辆，开通中心城区经八里湖新区至九江县公交线路，启动并分步推进庐山区乡村公交、环庐山公交。进一步健全以社会保险、社会救助、社会福利、社会慈善、社会养老为重点的社会保障体系，推进医疗、工伤、失业保险市级统筹，确保参保人数增长6%、基金征缴增长16%。加大对孤儿和城镇“三无”人员的救助帮扶力度。推进农村敬老院转型升级，探索社会化养老新途径，启动九江市养老服务中心建设，提高供养水平。加大医疗保障与救助力度，实行基本诊疗路径管理，推进全省异地看病同步结算，启动特困家庭大病统筹试点，开展农村贫困家庭妇女宫颈癌、乳腺癌免费救治，实现适龄儿童免费免疫规划预防接种全覆盖。巩固、完善、提升原有城郊蔬菜基地1.8万亩，新扩城郊蔬菜基地6000亩，新建、改造、提升中心城区农贸市场5个，完善农产品质量检测中心建设。改善农村群众生产生活条件，推进农村饮水一体化工程，解决25万农村人口饮水安全问题；继续在395个贫困村实施扶贫开发，推进农村扶贫帮扶到户工作，完成深山区、库区、农村污染区移民搬迁2万人。加大平安九江建设力度，启动中心城区“天网”工程四期和“封城计划”二期建设，新增高清探头300个。

　　协调发展社会事业。完善教育投入长效机制，严格落实财政教育经费法定增长要求。大力推进义务教育均衡化，积极发展学前教育，启动实施第二期学前教育三年行动计划，在中心城区建设4所省级示范幼儿园，完成九江小学八里湖校区、市中心幼儿园八里湖园区建设，优化中心城区湖滨片区义务教育网点布局，对全市410个农村义务教育学校进行标准化改造；加快普及高中阶段教育，规范发展民办教育，着力办好特殊教育，更加重视职业教育和高等教育发展。扎实开展农村文化三项活动，推进县级“三馆”、乡镇综合文化站建设和管理，抓好文化信息资源共享工程村级点建设，争创全国公共文化服务体系示范区。继续深入推进医药卫生体制改革，稳定新型农村合作医疗参保率，探索开展新农合大病医疗补充保险，将政策范围内住院费用支付比例提高到75%，统筹基金使用率提高到85%。抓好艾滋病、结核病、血吸虫等重大传染病防控，继续开展重性精神病等六项重大疾病免费救治，积极推进无偿献血工作。完善食品药品安全监管体系和检测能力建设，用严谨的标准、严格的监管、严厉的处罚、严肃的问责，确保食品安全。积极备战省运会，促进竞技体育、群众体育与体育产业加快发展。落实“单独两孩”生育政策，继续开展免费孕前优生健康检查，扩大免费婚前健康检查范围，实现农村计生纯女户社会养老保险全覆盖。扎实做好第三次全国经济普查工作。支持驻浔部队和国防后备力量建设，加强国防宣传教育，深入开展双拥共建，促进军地融合式发展。抓好民族宗教、外事侨务和对台工作，促进物价、审计、工商、质监、消防、防震减灾、人民防空、盐业、邮政、海事、地方志、档案、保密、编制、无线电管理、妇女儿童、老龄、残疾人、红十字和慈善等社会事业和谐发展。

　　加强和创新社会治理。建立健全突发事件预警和应急处置机制，提高应急处置能力和水平。健全信访工作制度，推行网上受理信访，及时就地解决群众合理诉求。建立涉法涉诉信访依法终结制度，把涉法涉诉信访纳入法治轨道解决。严格落实安全生产责任制，加强重点领域安全生产监管执法，深入开展隐患排查治理，坚决遏制重特大事故发生。完善社区和农村基层自治组织服务管理功能，加强流动人口和特殊人群管理服务。深入开展“六五”普法，继续推进法治九江建设，强化边界和谐共建，做好仲裁工作。积极构建立体化治安防控体系，严密防范和依法惩治违法犯罪活动，维护国家安全与公众安全。

　　三、加强政府自身建设

　　深入开展以“为民、务实、清廉”为主题的群众路线教育实践活动，切实转变政府职能，改进工作作风，不断提高政府的执行力、操作力和公信力，努力建设法治政府和服务型政府。

　　既要讲程序，又要重效率。按程序办事是政府工作的基本要求，提高工作效率是政府工作的关键所在。坚持依法行政，在市委的领导下，自觉接受人大及其常委会法律监督和政协民主监督，虚心接受社会舆论、新闻媒体和群众监督。认真办理人大代表建议和政协委员提案。严格按照法定程序和规章制度办事，推进政府工作的规范化、制度化和法制化。坚持合法高效，按照“工作要实、节奏要快、程序要到、效果要好”的原则，“快”字领先、“好”字当头、“实”字为要，转变工作作风，优化工作流程，不断提高行政效能。坚持抓具体，抓细节，抓关键，加强目标完成调度，强化工作过程监管，力促政府工作快节奏、高效率。

　　既要讲原则，又要重灵活。讲原则是政府依法行政的重要前提，重灵活是做好政府工作的有效途径。重灵活不是放弃原则，更不是上有政策下有对策，而是要注重研究政策，把握政策，用足用好用活政策，做到敢于担当，善于决断，勇于负责；而是要注重工作方法，做到敢攻坚、善破难，突出务实操作，创造性地开展工作，提高科学发展的能力和工作水平。

　　既要讲分工，又要重合作。成功的团队没有失败者，失败的团队没有成功者。坚持民主集中制，实行集体领导与分工负责相结合，做到各司其职，各负其责。重分工更重合作，牢固树立“一盘棋”思想，讲大局，谋全局，在合作中提升境界；加强沟通，密切配合，在合作中体现胸怀；真诚待人，和谐共事，在合作中锤炼品格。对市委市政府的决策部署，做到政令畅通，令行禁止，坚决杜绝推诿扯皮、办事拖沓，确保各项工作落到实处。

　　既要讲勤政，又要重廉政。勤政是国家工作人员的基本要求，廉政是各级干部的行为准则。坚持勤勉敬业，时刻牢记立党为公、执政为民的根本宗旨，把思想和精力集中到工作和事业上来。坚持廉洁从政，认真落实“一岗双责”，切实履行党风廉政建设责任制职责，模范遵守《廉政准则》，严格执行“八项规定”，坚决反对“四风”，厉行勤俭节约，严控“三公”经费和一般性开支，确保将有限的财力集中到惠民生、促发展上。推进政务公开，强化对行政审批、公共资源交易、行政执法及涉审中介组织等重点领域和关键环节的监管，让权力在阳光下运行，努力实现干部清正、政府清廉、政治清明。

　　各位代表，目标催人奋进，奋斗成就梦想！让我们紧密团结在以习近平同志为总书记的党中央周围，在省委、省政府和市委的正确领导下，凝心聚力，开拓创新，真抓实干，为九江争得应有地位、人民过上小康生活而努力奋斗！
